# Bougies with Alum

**Published:** 1845-03

**Authors:** 


### PRACTICAL MEDICINE, &c.

*Bougies with Alum.*—In diseases of the urethra, M. Jobert employs alum in the following manner. He spreads upon a table some burnt alum, reduced to powder. He then takes a wax bougie of a size suited to the dimensions of the canal, heats the end of it at a lamp, kneads it with his fingers, and then rolls the portion thus softened in the powder. This he continues till the bougie becomes thoroughly incorporated with it. When that is accomplished, the extremity of the bougie becomes white, and the superfluous alum is removed by a little friction. The instrument is then dipped in oil, introduced, and maintained for a time in the portion of the urethra where the patient complains of pain. By this means M. Jobert is enabled to employ an application which, from the tightness of the canal in certain cases, he could not have done by means of the ordinary *porte. caustique.*—*Annales de Thèrapeutique;* as quoted in *Jour. de Méd. et de Chirurgie Pratiques.*

